# Mandibular Radiographic Assessment in Sickle Cell Disease: A Systematic Review of Radiomorphometric Indices and Fractal Dimension

**DOI:** 10.1111/scd.70186

**Published:** 2026-05-12

**Authors:** Diego Belmiro do Nascimento Santos, Bruna Cristina Oliveira dos Santos, Larissa Conrado da Silva, Márcia Pereira Alves dos Santos, Luciana Munhoz, Bruno Augusto Benevenuto de Andrade, Jefferson R. Tenório

**Affiliations:** ^1^ Department of Pathology and Oral Diagnosis, School of Dentistry Universidade Federal do Rio de Janeiro Rio de Janeiro Rio de Janeiro Brazil; ^2^ Department of Forensic Dentistry and Public Health, School of Dentistry Universidade Federal do Rio de Janeiro Rio de Janeiro Rio de Janeiro Brazil; ^3^ Department of Stomatology, School of Dentistry Universidade de São Paulo São Paulo Brazil

**Keywords:** anemia, fractal analysis, hemoglobin SC disease, mandibular cortical index, sickle cell trait, sickle cell

## Abstract

**Aim:**

This systematic review aimed to investigate the association between sickle cell disease (SCD) and reduced bone mineral density (BMD) using radiomorphometric indices (RMI) and fractal dimension analysis (FDA) on dental radiographic images.

**Methods and Results:**

Observational studies evaluating BMD in individuals with SCD through RMI or FDA were included. A comprehensive search was conducted across six databases and grey literature in March 2025. Risk of bias was evaluated using the Joanna Briggs Institute Critical Appraisal Tool for cross‐sectional studies, and certainty of the evidence was assessed using the GRADE approach. Five cross‐sectional studies published between 2008 and 2024, conducted in Turkey and Brazil, were included. One hundred ninety‐nine SCD individuals were evaluated and the samples varied in terms of SCD genotype. The RMIs assessed were the mandibular cortical index, mandibular cortical width (MCW), panoramic mandibular index (PMI). FDA was applied in 03 studies. All studies had a risk of bias exceeding 25%, indicating low methodological quality.

**Conclusions:**

RMI and FDA show potential as adjunctive screening tools in patients with SCD. However, due to methodological heterogeneity and very low certainty of evidence, their clinical applicability remains limited

## 1 Introduction

Sickle cell disease (SCD) is a hereditary hemoglobinopathy caused by mutations in the β‐globin gene that result in the production of hemoglobin S (Hb S) [1]. Individuals who inherit one normal and one mutated allele are carriers of the sickle cell trait and are usually asymptomatic. In contrast, those who inherit two mutated alleles (such as in Hb SS, Hb SC, or Hb S–β‐thalassemia) develop sickle cell anemia [2]. In these individuals, red blood cells become rigid and acquire a sickle shape under low‐oxygen conditions, leading to hemolytic anemia, vascular occlusion/obstruction, chronic inflammation, and procoagulant state activation. These pathophysiological mechanisms contribute to the main clinical features of the disease, including chronic anemia, recurrent pain episodes, and progressive injury to multiple target organs [3, 4, 5].

SCD has a high global prevalence, predominantly affecting populations in the Mediterranean region, sub‐Saharan Africa, South Asia, South America, and the Middle East [6]. It is estimated that approximately 5% of the world population carries sickle cell allele, resulting in an average of 380,000 affected births each year [7, 8].

The clinical manifestations of with SCD may include episodes of fatigue, diffuse pain, pulmonary hypertension, priapism, acute chest syndrome, splenomegaly, and increased risk of stroke and nephropathies [9]. The disease also affects the oral and maxillofacial region, with reported findings such as mandibular osteomyelitis, aseptic pulp necrosis, atrophy of the lingual papillae, inferior alveolar nerve neuropathy, mucosal pallor, and delayed tooth eruption [1, 10]. In addition, patients may exhibit skeletal alterations in the maxillofacial complex, frequently presenting with Class II malocclusion, dental crowding, and an accentuated overjet [7].

SCD is usually diagnosed through newborn screening, employing hemoglobin electrophoresis and confirmatory genetic testing [11]. Management strategies include symptomatic treatment for pain episodes with analgesics, opioids, and nonsteroidal anti‐inflammatory drugs, as well as supportive therapies such as folic acid supplementation, hydroxyurea, alpha‐epoetin, blood transfusions, and in selected cases, hematopoietic stem cell transplantation [6, 12].

Reduced bone mineral density (BMD) is a well‐recognized complication in patients with hemoglobinopathies such as SCD, with prevalence rates estimated at 30%–50% in children and 60%–80% in adults [13, 14]. These individuals frequently present with cortical bone thinning and elevated fracture risk, which have been increased bone remodeling, chronic vitamin D deficiency, hormonal disturbances involving estrogen, testosterone, and parathyroid hormone, impaired osteoblast chemotaxis, and dysregulation of the RANK/RANKL/OPG signaling pathway [15]. A recent meta‐analysis of cross‐sectional, cohort, and case‐control studies reported a pooled BMD reduction prevalence of 57% (95% CI: 35%–80%) among individuals with SCD [1].

In this context, radiomorphometric indices (RMI) and fractal dimension analysis (FDA) have been employed in panoramic radiographs and other routine dental imaging modalities as screening tools for detecting reduced BMD. These methods enable the evaluation of cortical bone morphology by analyzing structural patterns and mineral loss, thereby contributing to diagnostic support within dental practice [16, 17]. Over time, numerous studies have demonstrated the utility of these indices in assessing BMD in diverse chronic systemic diseases associated with osteopenia or osteoporosis [18, 19, 20, 21] including other hemoglobinopathies [22]. Although these imaging‐based analyses do not replace dual‐energy X‐ray absorptiometry (DEXA) for precise BMD evaluation, they represent viable alternatives in settings where DEXA is limited by high costs or restricted accessibility [23].

Although maxillofacial bone alterations in SCD are clinically relevant to dental practice, there is still no consolidated overview of these findings in literature. Thus, considering the association between SCD and reduced BMD, this study investigates the following question: How are RMI and fractal dimension values affected in dental imaging of individuals with SCD?

## 2 Material and Methods

This study was registered in the International Prospective Register of Systematic Reviews (PROSPERO) under registration number CRD420251000134 and was conducted in accordance with the Preferred Reporting Items for Systematic Reviews and Meta‐Analyses (PRISMA) guidelines (http://www.prisma‐statement.org).

### 2.1 Eligibility Criteria

The inclusion criteria for this study were defined using PEO strategy. Studies were considered eligible if they involved human participants (Population) with SCD (Exposure) and assessed mandibular bone changes indicative of reduced BMD (Outcome). No restrictions were applied regarding age, sex, ethnicity, or disease stage. Eligible studies included observational research involving individuals with either cell anemia or sickle cell trait, in which osteoporotic‐like alterations were evaluated on routine dental imaging (panoramic radiographs or computed tomography) through RMI or texture analysis (FDA).

Studies were excluded if participants were using medications known to affect bone metabolism (e.g., antiresorptives, antiangiogenics, and corticosteroids), had a history of head and neck radiotherapy, other types of hemoglobinopathies, or had been diagnosed with metabolic bone disorders (e.g., osteopetrosis, Paget's disease, multiple myeloma, and osteogenesis imperfecta). Additionally, case reports, case series, literature reviews, letters to the editor, animal studies, laboratory‐based research, book chapters, and conference abstracts were excluded.

### 2.2 Search Strategy

A controlled vocabulary (MeSH terms), entry terms and free keywords were used in the search strategies, which were defined based on the systematic review research question. Searches were conducted in the PubMed/MEDLINE, Scopus, Web of Science, and Embase databases until March 2025. Grey literature was also searched using Google Scholar and OpenGrey. The search strategy included words related to “anemia, sickle cell,” “hemoglobin SC disease,” “sickle cell trait,” “jaw,” “mandibular cortical index,” and “fractal,” combined with Boolean operators AND/OR, without language or date restrictions (Supplementary 1). Monthly alerts were created to track recent publications on the topic. The reference lists of the included studies were also manually reviewed to identify additional relevant articles.

### 2.3 Study Selection

The articles identified through databases and manual searches were managed using the EndNote X7 software (Thomson Reuters, Philadelphia, Pennsylvania, USA), which enabled automatic removal of duplicates. The resulting records were exported to Rayyan (https://www.rayyan.ai), where remaining duplicates were manually reviewed and excluded. Two reviewers independently screened titles and abstracts using Rayyan following the predefined eligibility criteria. When the relevance of a study could not be determined by title and abstract, the full text was assessed. All studies selected in the initial screening were then evaluated in full to confirm inclusion. A third reviewer was consulted in cases of uncertainty regarding eligibility.

### 2.4 Data Collection

The following data were collected: author, year of publication, country of origin, study design, sample size (n), sex, age, SCD classification, image acquisition and analysis methos, and main findings.

### 2.5 Risk of Bias and Quality of Evidence

The risk of bias was assessed independently assessed by two reviewers using the Joanna Briggs Institute critical appraisal tool for cross‐sectional studies. Each item was marked as “Y” (yes), “N” (no), or “U” (unclear), with a greater number of “N” and “U” responses reflecting lower methodological quality. Disagreements were resolved by a third reviewer. Evidence quality was rated using GRADE tool (Grading of Recommendations Assessment, Development, and Evaluation), based on the study design, risk of bias, inconsistency, indirect evidence, imprecision, and sample size.

## 3 Results

### 3.1 Study Selection

**FIGURE 1:**
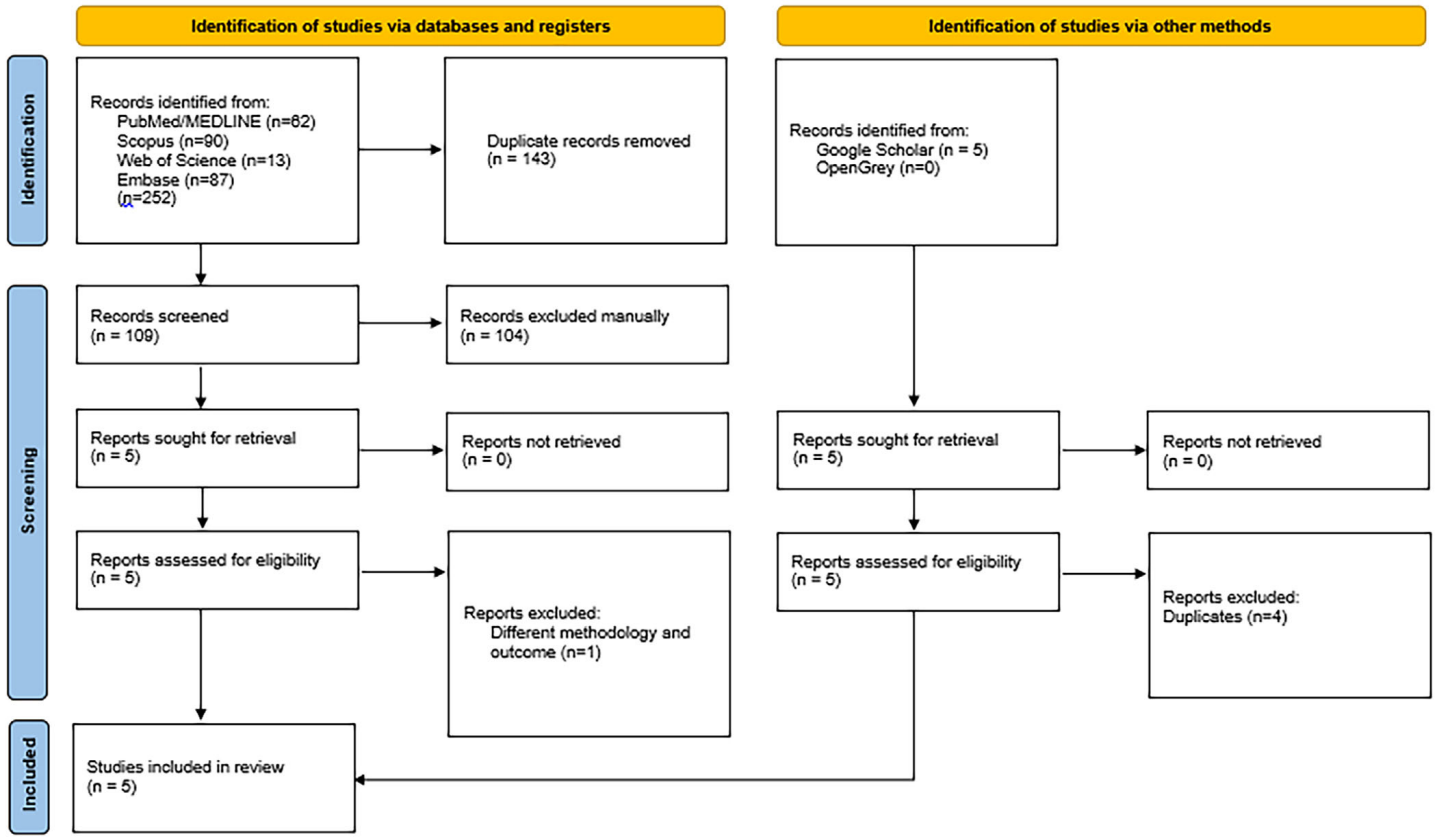
PRISMA flowchart.

A total of 252 articles was retrieved in the initial search. In the first stage, 143 duplicates were removed, leaving 109 articles. In the second stage, the titles and abstracts were reviewed, and 104 articles were excluded, resulting in 5 eligible studies [24, 25, 26, 27, 28]. Among these 5 studies, one study was excluded due to methodological inconsistencies [28]. The grey literature search identified five additional articles; four were excluded as duplicates, and only one met the inclusion criteria [29]. Thus, 5 studies were selected for data extraction and analysis in response to the review question (Figure 1).

### 3.2 Study Features Overview

The five articles included in this review were cross‐sectional studies [24, 25, 26, 27, 29] published between 2008 [24] and 2024 [29], conducted by research groups from Turkey [24, 26, 27] and Brazil [25, 29] (Table 1).

**TABLE 1:** Features of the selected studies: design, number of participants (*N*), sex, age, sickle cell disease genotype and diagnosis, methodology applied and main results.

Author (year), Country	Study design	*N*	Sex	Age	SCD data	Imaging exam	Methodology	Main results
Demirbas et al. (2008) [24], Turkey	Cross‐sectional	**SCD**: 35 **CG**: 26	**SCD** F: 14 M: 21 **CG** F: 10 M: 16	**SCD** < 20yo: 20 ≥ 20yo: 15 **CG** < 20yo: 18 ≥ 20yo: 8	Homozygous SCD	Digitized analog PR	FDA: a single ROI (in molar region) was used.	FD values were lower in SCD patients (mean 1.6855) than in CG (mean 1.7196) (*p* = 0.05).
Neves et al. (2012) [25], Brazil	Cross‐sectional	**SCD**: 44 **CG**: 34	UD	**SCD** < 40yo: 31 ≥ 40yo: 13 **CG** < 40yo: 12 ≥ 40yo: 22	SCD diagnosed by hemoglobin electrophoresis exam	Digitized analog PR	MCI, PMI, MI	C2 was most prevalent, especially in groups SCD< 40yo (*n* = 17) and CG ≥ 40yo (*n* = 13). Increased trabecular bone spacing was more frequent in SCD group. No significant differences in PMI and MI between SCD and CG individuals.
Serindere, Belgin (2019) [26], Turkey	Cross‐sectional	**SCD**: 30 **CG**: 30	**SCD** F: 16 M: 14 **CG** F: 14 M: 16	**SCD mean**: 34.47 **CG mean**: 38.67)	UD	Digital PR	MCW, PMI, FDA (ROI's: interdental, corpus and angulus)	MCW values were higher in the CG (median: 0.56) compared to the SCD group (median: 0.29) (*p*‐value unavailable). No significant differences between groups were observed for the other indices and FDA.
Temur et al., (2023) (27), Turkey	Cross‐sectional	SCD: 33 CG: 32	**SCD** F: 9 M: 24 **CG** F: 9 M: 23	Mean: 15.63 ± 2.49 (both groups)	20 individuals with homozygous (HbSS) and 13 with heterozygous (HbSB)	Digital PR	MCW, PMI, MCI, FDA (ROI's: 1 – angle, 2 – ramus, 3 –alveolar bone, and 4 – cortical bone.	FDA of ROI 1 and ROI 4 were significantly lower in the SCD group (*p* < 0.05). MCI categories did not differ between the SCD and CG (*p* > 0.05). PMI and MCW values were lower in the SCD group (*p*.< 0.05).
Almeida et al. (2024) [29], Brazil.	Cross‐sectional	SCD: 57 CG: 57	**SCD** F: 29 M: 28 **CG** F: 29 M: 28	**SCD mean**: 37.1 (18–59) **CG mean**: 37.2 (18–59)	SCD diagnosed by hemoglobin electrophoresis	CBCT, MDCT	Tb.Th, Tb.Sp, Tb.N, Po‐C.N, Po‐O, Po‐tot, and Conn.	Tb.Sp, Po‐O and Po‐tot values ​​were higher in the SCD group (*p* < 0.001). Tb.N and Po‐C.N values ​​were higher in the SCD group (*p*<0.001).

Abbreviation: SCD, sickle cell disease; CG, control group; F. female; M, male; PR, panoramic radiographs; FDA, fractal dimension analysis; ROI, region of interest; UD, unavailable data; MCI, mandibular cortical index; PMI, panoramic mandibular index; MI, mental index; MCW, mandibular cortical width; Tb.Th, thickness of the bone trabeculae, Tb.Sp, separation of the bone trabeculae; Tb.N, number of bone trabeculae; Po‐C.N, number of closed pores, Po‐O, frequency of open porosity; Po‐tot, frequency of total porosity, Conn, connectivity; CBCT, cone beam computed tomography; MDCT, multidetector computed tomography.

The studies included individuals of both sexes and various age groups, divided into homogeneous groups of SCD patients and healthy controls. Regarding SCD type, one study [24] included only homozygous individuals, another [27] included both homozygous and heterozygous individuals (HbSB), while three others [25, 26, 29] did not specify the SCD genotype. Concerning age, the study samples included participants younger than 20 years, older than 40 years, and others within this range. Raw age data were not available for most studies. For radiographic analysis, two studies [24, 25] used digitized analog panoramic radiographs, two studies [26, 27] employed digital panoramic radiographs, and one study [29] utilized cone‐beam computed tomography (CBCT) and multidetector CT scans. The RMI assessed included the mandibular cortical index (MCI) [25, 27], mandibular cortical width (MCW) [25, 26, 27] and panoramic mandibular index (PMI) [25, 26, 27]. Additionally, FDA was applied in three studies [24, 26, 27]. One study [29] evaluate additional parameters of mandibular bone microarchitecture, including: trabecular bone thickness (tb.th), trabecular bone separation (tb.Sp), trabecular bone number (tb.N), closed porosity number (Po‐C.N), open porosity frequency (Po‐O), total porosity frequency (Po‐tot), and connectivity (Conn).

### 3.3 Result of Individual Studies

In the study by Demirbas et al. [24], the mean FD values were slightly lower in the SCA group (1.6855) compared to healthy controls (1.7196). Greater variability was observed among SCA patients, with FD values ranging from 1.4217 to 1.8123 and a standard deviation of 0.0859, while the control group presented a narrower range (1.6381 to 1.8023) and lower variability (SD: 0.0459). Moreover, SCA patients under 20 years of age had significantly lower mean FD values compared to both older SCA patients and healthy controls (*p* < 0.05).

Neves et al. [25] evaluated the MCI, MCW, PMI, and qualitatively assessed trabecular spacing, characterized by reduced trabecular bone density and enlarged bone marrow spaces. No significant differences were observed between SCD patients and healthy controls for PMI and MCW. Regarding the MCI, the C2 classification (presence of semilunar cortical erosion) was the most prevalent, particularly among SCD individuals under 40 years of age (76.9%). Increased trabecular spacing was also more frequently observed in the SCD group.

In the study by Serindere and Belgin [26], the sample was homogeneous (SCD and control groups) with respect to age and sex. FDA was performed in three regions of interest (ROIs): mandibular angle, mandibular body, and interdental bone. No significant differences were observed among the ROIs (*p* > 0.05). The PMI and MCW also showed higher median values in the control group compared to the SCD group, although statistical significance was not reported.

Temur et al. [27] evaluated FD in four ROIs: the mandibular angle (ROI 1), mandibular body (ROI 2), alveolar bone (ROI 3), and cortical bone (ROI 4). No significant differences in age or sex were observed between the groups. FD values were significantly lower in the SCD group for ROI 1 and ROI 4 (*p* < 0.05). In addition to FDA, PMI and MCW values were also significantly lower in the SCD group (*p* < 0.05), suggesting reduced BMD. However, no significant difference was found in MCI values between groups (*p* > 0.05).

Almeida et al. [29] was the only study to use three‐dimensional imaging to evaluate bone microarchitecture. The sample was homogeneous in terms of sex, with a mean age of 37 years. The SCD group exhibited significantly higher values for tb.Sp, Po‐O, Po‐tot, tb.N, and Po‐C.N (*p* < 0.001), indicating increased trabecular spacing, reduced trabecular number, and increased bone porosity. Although connectivity, which reflects the degree of trabecular interconnection and potential bone strength, was lower in the SCD group, the difference was not statistically significant (*p* = 0.24).

### 3.4 Risk of Bias

All included studies presented a risk of bias greater than 25%, reflecting limited methodological quality (Table 2). None of the five studies adequately identified or addressed potentially confounding factors, nor did they distinguish between different SCD subtypes. Relevant variables such as medication use, alcohol intake, smoking habits, and postmenopausal status in women were largely unreported. In 60% of the studies [24, 26, 29] no specification was made regarding the type of SCD. Moreover, the gold standard diagnostic method (hemoglobin electrophoresis) was not mentioned in three studies [24, 26, 29]. Intra‐ and inter‐observer reliability were also not clearly reported in these same studies, further limiting the strength of their findings.

**TABLE 2:** Risk of bias of the included studies (Joanna Briggs Institute Critical Appraisal Checklists for cross‐sectional studies).

	Dermibas et al., 2008 [24]	Neves et al., 2012 [25]	Serindere, Belgin, 2019 [26]	Temur et al., 2023 [27]	Almeida et al., 2024 [29]
Q1 Were the criteria for inclusion in the sample clearly defined? 0	Y	Y	Y	Y	Y
Q2 Were the study subjects and the setting described in detail? 0	Y	Y	Y	Y	Y
Q3 Was the exposure measured in a valid and reliable way? 3	N	Y	U	Y	N
Q4 Were objective standard criteria used for measurement of the condition? 3	U	Y	N	U	Y
Q5 Were confounding factors identified? 5	N	N	N	N	N
Q6 Were strategies to deal with confounding factors stated? 5	N	N	N	N	N
Q7 Were the outcomes measured in a valid and reliable way? 1	Y	Y	Y	Y	N
Q8 Was appropriate statistical analysis used? 3	N	Y	N	Y	N
Overall risk	62.5%	25%	62.5%	37.5%	62.5%

*Note*: Y = yes; N = no; U = unclear. The number after each of the 8 items in the checklist indicates the number of studies receiving an assessment of “Not” or “Unclear”. The overall risk of bias is calculated as the percentage of the 8 items in the checklist receiving an assessment of “Not” or “Unclear” for each of the 05 studies.

### 3.5 Quality of Evidence

As emphasized in the previous section, the studies demonstrated low methodological quality, which contributed to the overall classification of the evidence as very low. While inconsistency was not considered a major issue in the five included studies, there was high degree of imprecision, primarily due to the small number of events: less than 300 patients for dichotomous variables (MCW and MCI) and fewer than 400 for continuous variables (FDA, MPI, and MI). The evidence was affected by indirectness, as the studies did not represent the full clinical spectrum of SCD. These factors, combined along with the exclusively cross‐sectional design of all included studies, further lowered the certainty of the evidence (Table 3).

**TABLE 3:** Certainty of evidence (GRADEpro tool).

Certainty assessment						No. of patients	
No. of studies	Study design	Risk of bias	Inconsistency	Indirectness	Imprecision	Sickle cell disease	Certainty
5	non‐randomized studies	very serious^a^	not serious	serious^b^	very serious^c^	199	⨁◯◯◯ Very low^a^, ^b^, ^c^

^a^ All studies presented a risk of bias ≥ 25%.

^b^ It is not possible to state that all the main subtypes of sickle cell disease were analyzed in the studies.

^c^ The number of events was < 300 for dichotomous variables and < 400 for continuous variables.

## 4 Discussion

Well‐designed clinical studies should be done in order to better qualify the dental management of people with SCD. Assessing BMD in individuals with SCD is clinically relevant, given the wide range of complications associated with both BMD alterations and SCD itself, factor that markedly impact patients’ quality of life. In this context, the evaluation of RMI and FDA in dental imaging has proven beneficial not only in disorders directly affecting bone metabolism [19, 30], but also in hematological conditions such as hemophilia [31] and thalassemias [22]. Based on this issue, the present study systematically reviewed available evidence on the application of these tools for screening low BMD in individuals with SCD and found that there is a weak evidence and a low level of recommendation.

There is consistent evidence suggesting the use of RMI and FDA for BMD screening. A systematic review by Kinalski et al. [32] reported that the MCI, as observed on panoramic radiographs, has high sensitivity (0.81, 95% CI, 0.78–0.84) for detecting at least osteopenia in women over 30 years old. Similar findings have been reported by other authors using the same index and panoramic radiographs [33]. The MCI has also been evaluated with CBCT, demonstrating good correlation with vertebral and femoral BMD values measured by DEXA [34, 35].

Interestingly, some studies have reported the capability of dental imaging to identify bone alterations in individuals with SCD and to correlate these findings with systemic predictors of disease severity. Neves et al. [36] examined a sample of 71 individuals with SCD (HbSS and HbSC genotypes) and found an association between increased mandibular trabecular spacing and a history of jaundice, as well as between horizontally oriented trabecular bone in the molar region and a history of stroke. These findings suggest a possible connection between mandibular bone alterations and systemic complications in individuals with SCD.

Osteoporotic changes in individuals with SCD are multifactorial, and certain risk factors must be considered in the assessment of these patients, including vitamin D deficiency, bone marrow hyperplasia, and iron overload. None of the studies included in this review investigated these variables in detail within SCD populations. However, Hamdy et al. [37] examined the role of vitamin D deficiency in 80 individuals with SCD under the age of 20, including 59 with the HbSS genotype and 21 with HbSβ. The study reported skeletal alterations in 60% of the SCD patients, compared to 26.7% in the control group, and identified a statistically significant correlation between vitamin D levels and biomarkers of hemolysis [37]. In a related study, Mahachoklertwattana et al. [38] analyzed cellular and structural bone changes in patients with β‐thalassemia and found that elevated iron levels were linked to local iron deposition, inhibition of bone mineralization, and impaired osteoblast maturation. Furthermore, increased erythropoiesis activity associated with bone marrow hyperplasia may interfere with the proliferation, differentiation and maturation of osteogenic cells [38]. These mechanisms should be considered in future research to strengthen the interpretation of BMD findings in SCD populations.

The PMI and MCW were the most frequently used RMI among the studies reviewed [25, 26, 27]. In the present review, only one study reported that the PMI may be a useful screening tool for BMD in individuals with SCD [27]. While this finding is pertinent, it is not entirely unexpected. The PMI is calculated as the ratio between the cortical width at the mental foramen and the distance from the mandible inferior lower border to the lower margin of the mental foramen [39]. Given the dependence on precise anatomical landmarks, this measurement requires strong intra‐ and interobserver agreement to ensure reliability. However, the study by Temur et al. [27] did not clearly report whether intra‐ and interobserver reliability analyses were conducted for PMI. In contrast, Neves et al. [25] found no statistically significant differences in PMI values between SCD patients and healthy controls but reported high interobserver agreement (ICC = 0.91). Overall, the estimated sensitivity and specificity of the PMI for identifying low BMD, based on a threshold of ≤ 0.32, are 0.723 (SE 0.160; 95% CI, 0.352–0.926) and 0.733 (SE 0.066; 95% CI, 0.587–0.841), respectively [39]. For MCW the location of the mental foramen is determined by drawing a line tangent to the level of the foramen and another perpendicular; the distance from the point of intersection between the lines to the lower border of the mandible is measured. In Serindere [26] the median was considerably lower in the SCD group (0.29) compared to the control group (0.56), although the p‐value was not reported, similar to Temur [27] (*p* < 0.05), with both suggesting the mandibular cortical reduction associated with SCD. The result differs from Neves [25], who did not find statistically significant values ​​using this index. Still, it is important to emphasize that linear measurements on panoramic radiographs are highly operator‐dependent, as patient mispositioning may distort image and affect measurement accuracy.

Our analysis identified that FDA was employed in three studies [24, 26, 27], two of which reported statistically significant findings suggesting its potential for detecting mandibular bone alterations. However, these results should be interpreted with caution. Definitions of ROIs varied considerably among studies: one assessed a single ROI in medullary bone [24], while only one included a ROI the mandibular base [27]. Franciotti et al. [40], in their meta‐analysis, previously raised concerns about the reliability of FDA in distinguishing individuals with osteoporosis from healthy controls. They attributed these limitations to marked heterogeneity among studies and emphasized the need for methodological standardization. Issues noted were similar to those identified in the present review, including variation in ROI size, shape, and location; variations in image processing protocols (which can affect inherent magnification and distortion); differing algorithms applied to calculate fractal dimension; and inconsistent results when comparing cortical versus trabecular bone structures.

The main limitations of this systematic review are the overall low methodological quality of the included studies. Small sample sizes, cross‐sectional designs, and marked methodological heterogeneity weakened the external validity of the findings, complicated data interpretation, and interfere the establishment of causal inferences. Moreover, main clinical variables (e.g. nutritional status [15], the presence of race/ethnicity groups [41], type and severity of SCD, smoking status, alcohol consumption, postmenopausal status) were either poorly reported or entirely omitted in most studies. This lack of detailed clinical information restricts the ability to reliably attribute reduced BMD specifically to SCD. Finally, despite multiple attempts to contact corresponding authors to access to raw data, it was not possible to perform a quantitative synthesis of the RMI and FDA results through meta‐analysis.

## 5 Conclusions

In summary, the PMI, MCW, and FDA are the most commonly employed methods for assessing reduced BMD in individuals with SCD. However, findings from different studies remain inconsistent, primarily due to methodological heterogeneity and a high risk of bias. These limitations contribute to overall very low certainty of the evidence. Future investigations should prioritize well‐designed, adequately powered studies that incorporate detailed clinical data to support the validation and clinical applicability of RMI and FDA in this population.

## Author Contributions

J.R.T. study conception and design. D.C.N.S., B.C.O.S., and J.R.T. development of the review protocol and search strategy. D.C.N.S. and B.C.O.S. literature search, study selection, and data extraction. L.C.S. and B.A.B.A. methodological quality assessment. M.P.A., L.M., and J.R.T. data interpretation and critical scientific input. B.A.B.A. and J.R.T. project coordination and supervision. DCNS, B.C.O.S., L.C.S., and L.M. manuscript drafting. M.P.A., L.M., B.A.B.A., and J.R.T. manuscript critical revision and final approval.

## Funding

This research did not receive any specific grant from funding agencies in the public, commercial, or not‐for‐profit sectors.

## Patient consent

No patient consent required.

## Conflicts of Interest

The authors declare that they have no conflict of interest.

## Supporting information




**Supporting File 1**: scd70186‐sup‐0001‐SuppMat.docx
