# Supplementary material for: Mandibular Radiographic Assessment in Sickle Cell Disease: A Systematic Review of Radiomorphometric Indices and Fractal Dimension
Source: Spec Care Dentist. 2026 May 12;46:e70186. doi: 10.1111/scd.70186 (PMC13162086; doi:10.1111/scd.70186)
Supplement: Supplementary file 1 — Supporting File 1: scd70186‐sup‐0001‐SuppMat.docx [file SCD-46-0-s001.docx]

| DATABASE | SEARCH |
| --- | --- |
| Pubmed/MEDLINE | ("anemia, sickle cell"[MeSH Terms] OR "anemia sickle cell"[Title/Abstract] OR "acute chest syndrome"[MeSH Terms] OR "acute chest syndrome"[Title/Abstract] OR "hemoglobin sc disease"[MeSH Terms] OR "hemoglobin sc disease"[Title/Abstract] OR "sickle cell trait"[MeSH Terms] OR "sickle cell trait"[Title/Abstract] OR "vaso occlusive crises"[Title/Abstract]) AND ("jaw"[MeSH Terms] OR "mandible"[MeSH Terms] OR "trabecular pattern"[Title/Abstract] OR "mandibular cortex"[Title/Abstract] OR "mandibular cortical index"[Title/Abstract] OR "fractal"[Title/Abstract] OR "mental index"[Title/Abstract]) |
| Scopus | (INDEXTERMS ("anemia, sickle cell" OR "acute chest syndrome" OR "hemoglobin SC disease" OR "sickle cell trait") AND INDEXTERMS ("jaw" OR "mandible"))  OR  (TITLE-ABS-KEY ("anemia sickle cell" OR "acute chest syndrome" OR "hemoglobin SC disease" OR "sickle cell trait" OR "vaso occlusive crises") AND TITLE-ABS-KEY ("trabecular pattern" OR "mandibular cortex" OR "mandibular cortical index" OR fractal OR "mental index")) |
| Web of Science | TS=("anemia, sickle cell" OR "anemia sickle cell" OR "acute chest syndrome" OR "hemoglobin SC disease" OR "sickle cell trait" OR "vaso occlusive crises")  AND  TS=("jaw" OR "mandible" OR "trabecular pattern" OR "mandibular cortex" OR "mandibular cortical index" OR "fractal" OR "mental index") |
| Embase | ('anemia, sickle cell'/exp OR 'acute chest syndrome'/exp OR 'hemoglobin sc disease'/exp OR 'sickle cell trait'/exp OR 'anemia sickle cell':ab,ti OR 'acute chest syndrome':ab,ti OR 'hemoglobin sc disease':ab,ti OR 'sickle cell trait':ab,ti OR 'vaso occlusive crises':ab,ti) AND ('jaw'/exp OR 'mandible'/exp OR 'fractal analysis'/exp OR 'mandibular cortical index':ab,ti OR 'trabecular pattern':ab,ti OR 'mandibular cortex':ab,ti OR 'mental index':ab,ti) |
| Google Scholar | "anemia, sickle cell" AND "mandibular cortex"  "anemia, sickle cell" AND "mandibular cortical index"  "anemia, sickle cell" AND “fractal" |
| OpenGrey | "anemia, sickle cell" AND "mandibular cortex"  "anemia, sickle cell" AND "mandibular cortical index"  "anemia, sickle cell" AND “fractal" |

**Supplementary 1** – Search strategies.
